# Correlation between CXCR4, CXCR5 and CCR7 expression and survival outcomes in patients with clinical T1N0M0 non‐small cell lung cancer

**DOI:** 10.1111/1759-7714.13645

**Published:** 2020-09-08

**Authors:** Zhao Yue, Ding Ningning, Yang Lin, Ying Jianming, Zhang Hongtu, Yuan Ligong, Li Feng, Wang Shuaibo, Mao Yousheng

**Affiliations:** ^1^ Department of Thoracic Surgery, National Cancer Center/National Clinical Research Center for Cancer/Cancer Hospital Chinese Academy of Medical Sciences and Peking Union Medical College Beijing China; ^2^ Department of Pathology, National Cancer Center/National Clinical Research Center for Cancer/Cancer Hospital Chinese Academy of Medical Sciences and Peking Union Medical College Beijing China

**Keywords:** CCR7, CXCR4, CXCR5, non‐small cell lung cancer, survival outcome

## Abstract

**Background:**

Lung cancer is the leading cause of cancer‐related death. Even if early detection and treatment have proven to be effective, the survival outcomes are still poor.

**Methods:**

Tissue samples and clinicopathological data of 244 patients with clinical T1N0M0 NSCLC were collected. We investigated CXCR4, CXCR5 and CCR7 expression levels using the immunohistochemical method and analyzed their correlations with clinicopathological characteristics and survival outcomes.

**Results:**

Elevated expression levels of CXCR4, CXCR5 and CCR7 were found in tumor tissues (*P* < 0.001). The expression levels were remarkably different in histological type (CXCR4, *P* = 0.032; CXCR5, *P* < 0.001; CCR7, *P* < 0.001) and LVI (CXCR4, *P* = 0.017; CXCR5, *P* = 0.030; CCR7, *P* < 0.001). In addition, CXCR4 and CXCR5 expression were significantly different in tumor differentiation (CXCR4, *P* < 0.001; CXCR5, *P* < 0.001). Survival analysis showed that patients with positive CXCR4 expression had a significantly lower five‐year DFS (*P* = 0.007) and a lower five‐year OS (*P* = 0.010). Patients in the CXCR5 positive group had a significantly lower five‐year DFS (*P* = 0.038) and a lower five‐year OS (*P* = 0.220), which were statistically insignificant. However, five‐year DFS and five‐year OS of patients with positive CCR7 expression were significantly higher (DFS: *P* < 0.001; OS: *P* < 0.001). CXCR5 and CCR7 expression were found to be independent prognostic factors through multivariate analysis.

**Conclusions:**

Expression levels of CXCR4, CXCR5 and CCR7 were significantly higher in tumor tissues, and expression of CXCR5 and CCR7 were independent prognostic factors for survival. Moreover, all three chemokines were correlated to the survival outcomes of patients with clinical T1N0M0 NSCLC, providing potential prognosticators and therapy targets for lung cancer treatment.

## Graphical abstract

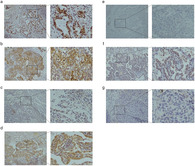

## Introduction

Lung cancer is now the leading cause of cancer‐related death in both China and worldwide.^1^, ^2^ Histologically, lung cancer is classified into non‐small cell lung cancer (NSCLC), which accounts for approximately 85% of all lung cancers, and small cell lung cancer (SCLC), accounting for the remaining 15%.^3^ Despite advances in screening, diagnosis and multidisciplinary treatments in recent years, the survival outcomes of patients with lung cancer are still not satisfying. Since early symptoms are neither specific nor conspicuous, many patients are diagnosed at advanced stages with poor prognoses. Fortunately, early detection with low‐dose computed tomography (LDCT) has been reported to be effective and can reduce lung cancer mortality up to 20% among high risk individuals.^4^, ^5^ However, the five‐year survival of all stages is only 18%.^6^ Thus, apart from LDCT screening, following precise treatments are also needed to further improve survival outcomes of patients with lung cancer. Previous studies have demonstrated that recurrence or metastasis occurs in approximately 20% of patients with early stage NSCLC, even though surgeries had been done, which indicated that there were other factors that impacted on the prognosis of patients with early NSCLC other than TNM stage. Thus, finding the prognostic factors is of great significance to identify high‐risk patients and guide individualized precise treatment to reduce recurrence and improve survival outcomes.

Chemokines are a superfamily of small (8–10 kDa) proteins that can cause the directed migration of certain subsets of leukocytes and are induced by inflammatory cytokines, growth factors and pathogenic stimuli.^7^, ^8^, ^9^ Chemokines are the major regulators of cell trafficking and adhesion in vivo.^10^ Based on the configuration of the first two conserved cysteine residues adjacent to the N‐terminal region, chemokines are classified into four subfamilies (C, CC, CXC and CXXXC families).^8^ Chemokine receptors are a superfamily of seven transmembrane spanning proteins coupled to G‐protein‐coupled receptors (GPCRs). Most of these receptors bind to more than one chemokine. Their binding leads to conformational changes, following activation of different signaling pathways, which mediate different biological processes, and finally results in tumor angiogenesis, growth, epithelial mesenchymal transition (EMT), and further metastases.^11^, ^12^, ^13^

Many kinds of chemokine ligands and receptors have been reported to be overexpressed in different types of tumors.^14^, ^15^, ^16^, ^17^, ^18^, ^19^ Emerging evidence revealed that elevated expression levels of CXCR4, CXCR5 and CCR7 were relevant to tumorigenesis, cell growth, survival, and site‐specific metastasis.^20^, ^21^, ^22^, ^23^, ^24^, ^25^ CXCR4, also known as CD184 or fusin, is a specific chemokine receptor for CXCL12. CXCR4 was initially cloned from leukocytes,^26^ but now, its expression has been found in different types of cells and plays an important role in cell migration.^8^ Recently, it has been reported that CXCR4 is overexpressed and plays an important role in the survival, proliferation, migration and metastasis of different kinds of tumors, such as breast, gastric, esophageal, and prostate cancers, etc.^22^, ^25^, ^27^, ^28^ CXCR5 was first isolated from Burkitt lymphoma and named as Burkitt's lymphoma receptor 1 (BLR1),^29^ and it is highly expressed on mature recirculating B‐lymphocytes, a subpopulation of follicular helper T cells (T_FH_) and skin‐derived migratory dendritic cells (DCs), and controls their migration into secondary lymphoid organs with the gradient of its ligand, CXCL13.^30^, ^31^, ^32^ The CXCL13/CXCR5 axis is involved in the progression of many hematological and solid malignancies.^20^, ^21^ CCR7 is mainly expressed on naïve B and T cells, some central memory T cells and mature DCs and mediates lymphocyte migration and homing to secondary lymphoid organs.^33^, ^34^, ^35^ Aberrant expression of CCR7 has been identified in several types of tumors, including breast cancer, head and neck squamous cell carcinoma, esophageal squamous cell carcinoma and melanoma.^23^, ^24^, ^36^, ^37^, ^38^ Some studies have also revealed the roles of CXCR4, CXCR5 and CCR7 in lung cancer.^39^, ^40^, ^41^, ^42^ However, the correlation of these chemokine receptors with survival outcomes has not yet been clearly described.

To further investigate the correlation between CXCR4, CXCR5 and CCR7 expression and survival outcomes in patients with clinical T1N0M0 NSCLC, we collected tumor and corresponding normal tissues, together with clinicopathological data of patients with clinical T1N0M0 NSCLC who underwent curative lobectomies with systematic lymph node dissections, detected expression levels of CXCR4, CXCR5 and CCR7 in tumor and corresponding normal tissues using the immunohistochemical method and then analyzed their correlation with survival outcomes, in order to evaluate the potential prognostic roles of CXCR4, CXCR5 and CCR7 in patients with clinical T1N0M0 NSCLC.

## Methods

### Patients

From January 2011 to January 2012, clinicopathological data of patients with clinical T1N0M0 NSCLC who underwent curative lobectomy with systematic lymph node dissection at the Department of Thoracic Surgery, National Cancer Center/National Clinical Research Center for Cancer/Cancer Hospital, Chinese Academy of Medical Sciences and Peking Union Medical College, Beijing, China, was retrospectively collected. The inclusion criteria were as follows: ≤ 75 years old; no history of other malignancies; no previous antitumor treatment (chemotherapy, radiotherapy, immunotherapy, etc.); and underwent curative lobectomy with systematic lymph node dissection. The eighth edition of lung cancer stage classification by the AJCC/UICC was used to determine the TNM stages. Finally, 244 patients were enrolled into the study.

### Tumor specimens

The tumor and its corresponding normal (remote nonneoplastic lung) tissues were fixed in 10% (v/v) formalin immediately after resection. After being embedded in paraffin, the specimens were prepared in serial sections of 5 μm and reserved for hematoxylin‐eosin (HE) staining. The slides were then examined by professional pathologists to determine the pathological type and existence of lymphovascular invasion (LVI) of the tumor.

### Immunohistochemistry

We performed immunohistochemistry (IHC) for CXCR4, CXCR5 and CCR7 on 244 NSCLC tumor and their corresponding normal samples, which were set to be the control group. The slides were deparaffinized in xylene (I, II and III) for 10 minutes each, then rehydrated through ethanol of different gradients (100%, 85% and 75%) for 10 minutes each and washed in deionized water and phosphate‐buffered saline (PBS) (ZhongShanJinQiao, Beijing, China). Antigen retrieval was implemented by incubation with 0.01 M sodium citrate buffer (pH = 6.0) in boiling water for 20 minutes and then cooled down to room temperature, and the activity of endogenous peroxidase was blocked by incubation with 3% hydrogen peroxide (H_2_O_2_) for 10 minutes. PBS washing was applied after each step for four times. After incubation in a nonspecific stain blocking agent, slides were incubated for two hours at 37°C with the following primary antibodies: anti‐human CXCR4 antibody (GR262216‐26; Abcam, Cambridge, UK; 1:100), anti‐human CXCR5 antibody (GR297692‐6; Abcam, Cambridge, UK; 1:100), and anti‐human CCR7 antibody (GR314167‐1; Abcam, Cambridge, UK; 1:100). This was followed by incubation in the PV‐9000 Polymer Detection System (ZhongShanJinQiao, Beijing, China), which contained the PV 9000 Kit Polymer Helper and PV 9000 Kit polyperoxidase‐anti‐mouse/rabbit IgG, for 20 minutes at 37°C and then washing with PBS for three times. After incubation, the slides were stained with a 3, 3′‐diaminobenzidine (DAB) (ZhongShanJinQiao, Beijing, China) as a chromogen. Counterstaining was implemented with hematoxylin (ZhongShanJinQiao, Beijing, China). Subsequently, slides were washed with water and ammonia, dehydrated in 75%, 85%, and absolute alcohols for five minutes each and sealed with resin before evaluation.

To analyze the immunohistochemical staining of CXCR4, CXCR5 and CCR7, slides were evaluated by two professional pathologists who were blind to each other. The intensity of staining was evaluated and graded from 0 to 3, with 0 for no staining (negative), one for light yellow (weak), two for yellow (moderate) and three for brown (strong). The numbers of positively stained cells were scored as follows: 0, ≤ 25%; 1, 25%–50%; and 2, 51%–100%. The two values obtained were multiplied to calculate a final score (maximum value, six). Samples were classified into negative (score < 2) or positive (score ≥ 2) for further analysis.

### TCGA data mining

We performed data mining to compare the expression levels of CXCR4, CXCR5 and CCR7 in lung adenocarcinoma (LUAD) and lung squamous cell carcinoma (LUSC) in TCGA database through AIPuFu (http://aipufu.com). The expression of each chemokine in paired tumor and normal tissues is shown in boxplots.

### Statistical analysis

The clinicopathological characteristics were compared between the groups using Student's *t*‐tests for continuous variables with normal distributions, Mann‐Whitney U tests for continuous variables with abnormal distributions, and chi‐square tests for categorical variables. OS was defined as the period between the date of surgery and death due to any cause or the last follow‐up. DFS was defined as the period after successful treatment during which there were no signs or symptoms of the disease that was treated. Both the cumulative OS and DFS rates were estimated by the Kaplan‐Meier method, and the differences were compared between groups by the log‐rank test. To identify prognostic factors, univariate and multivariate analyses were performed using the Cox proportional hazards regression model. Baseline variables that were considered clinically relevant or that showed a univariate relationship with the outcome were incorporated into a multivariate Cox proportional hazards regression model. Variables for inclusion were carefully chosen, given the number of events available, to ensure parsimony of the final model.

All statistical analyses were performed using IBM SPSS software version 24.0 (IBM Corp., Armonk, NY, USA) and GraphPad Prism 8 (GraphPad Software, San Diego, CA, USA). The results were considered statistically significant when the *P*‐value was less than 0.05.

## Results

### Patients' clinicopathological characteristics

A total of 244 qualified patients who were diagnosed with clinical T1N0M0 NSCLC were enrolled into this study, and their clinicopathological characteristics are shown in Table 1. There were 138 male patients and 106 female patients, with a median age of 59 years old. In total, 189 patients were diagnosed with LUAD, 50 patients with LUSC and five patients with other types of lung cancer. The median tumor size was 2.0 cm. After surgery, pathological examinations showed that 181 patients were free of lymph node metastasis, 28 patients had N1 metastasis and 35 patients had N2 metastasis. A total of 37 patients were diagnosed with well differentiated tumors, 131 with moderately differentiated tumors and 76 with poorly differentiated tumors. LVI was diagnosed in 96 patients during pathological examination.

**Table 1:** Clinicopathological characteristics of patients

Variable	Patients (*n* = 244)
Sex, n (%)	
Male	138 (56.6%)
Female	106 (43.4%)
Median age (range), year	59 (37–74)
Median tumor size (range), cm	2.0 (1.1–3.0)
Tumor location, n (%)	
RUL	81 (33.2%)
RML	11 (4.5%)
RLL	42 (17.3%)
LUL	73 (29.9%)
LLL	35 (14.3%)
Main bronchus	2 (0.8%)
Clinical T stage	
T1b	128 (52.5%)
T1c	116 (47.5%)
Pathological T stage	
T1b	88 (36.1%)
T1c	65 (26.6%)
T2a	91 (37.3%)
Pathological N stage, n (%)	
N_0_	181 (74.2%)
N_1_	28 (11.5%)
N_2_	35 (14.3%)
Histological type, n (%)	
Adenocarcinoma	189 (77.5%)
Squamous cell carcinoma	50 (20.5%)
Others	5 (2.0%)
Histological subtype, n (%)*	
Lepidic predominant	18 (9.5%)
Acinar predominant	71 (37.5%)
Papillary predominant	48 (25.4%)
Solid predominant	25 (13.3%)
Micropapillary predominant	27 (14.3%)
Tumor differentiation, n (%)	
Well	37 (15.2%)
Moderate	131 (53.7%)
Poor	76 (31.1%)
Lymphovascular invasion, n (%)	
Yes	96 (39.3%)
No	148 (60.7%)

LLL, left lower lobe; LUL, left upper lobe; RLL, right lower lobe; RML, right middle lobe; RUL, right upper lobe.

### Expression of CXCR4, CXCR5 and CCR7 in tumor and normal tissues

**Figure 1:**
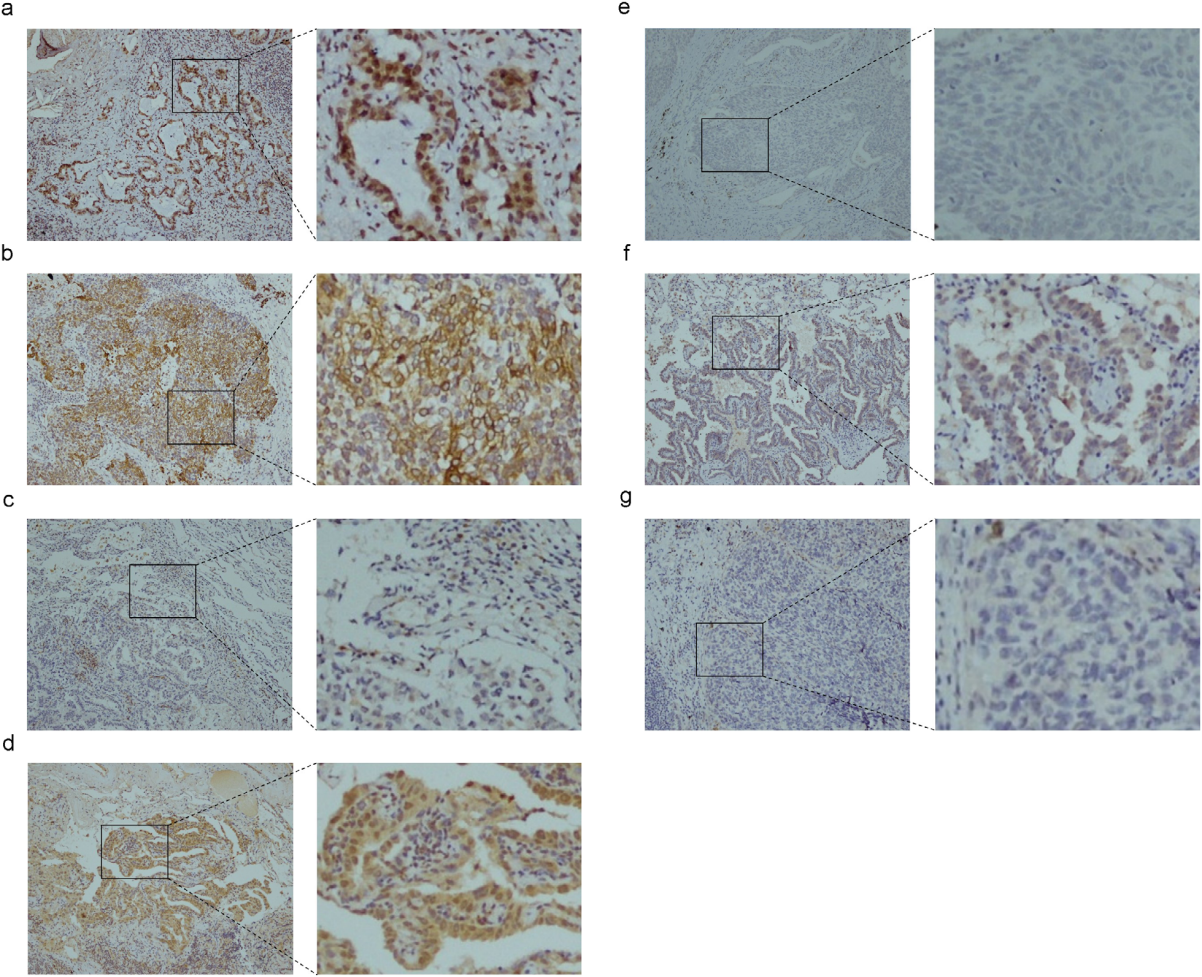
Immunohistological results of CXCR4, CXCR5 and CCR7 expression in tumor and corresponding normal tissues. (**a**–**c**) Expression of CXCR4 on membrane and nucleus (**a**); on membrane only (**b**); and negative expression (**c**). (**d**–**e**) Positive (**d**); and negative (**e**) expression of CXCR5. (**f**–**g**) Positive (**f**); and negative (**g**) expression of CCR7.

Fig 1 shows the expression of CXCR4, CXCR5 and CCR7 in tumor and corresponding normal tissues. CXCR4 was found to be mainly expressed on the membrane, while nuclear expression was also found in some cases (Fig 1a and b). CXCR5 and CCR7 expression were found on the membrane and in the cytoplasm of tumor cells (Fig 1d and f). Positive CXCR4 expression was found in 195 (79.9%) samples of tumor tissues and 12 (4.9%) samples of normal tissues, while positive cases for CXCR5 and CCR7 were 155 (63.5%) and 117 (48.0%) samples of tumor tissues and 15 (6.1%) and six (2.5%) samples of normal tissues, respectively (Table 2). CXCR4 had the highest positive rate, and all these differences were significant (*P* < 0.001). Among CXCR4 positive cases, 176 had membrane expressions only and 19 had both membrane and nuclear expressions.

**Table 2:** Expression of CXCR4, CXCR5 and CCR7 in tumor and corresponding normal tissues

Variable	Tumor tissue	Normal tissue	*P*‐value
CXCR4	195 (79.9%)	12 (4.9%)	<0.001
CXCR5	155 (63.5%)	15 (6.1%)	<0.001
CCR7	117 (48.0%)	6 (2.5%)	<0.001

### Correlation between CXCR4, CXCR5 and CCR7 expression and clinicopathological characteristics

We then evaluated the correlation between CXCR4, CXCR5 and CCR7 expression and clinicopathological characteristics, and the results are shown in Table 3. Patients were classified into two groups depending on the chemokine expression in tumor tissues.

**Table 3:** Correlation between CXCR4, CXCR5 and CCR7 expressions and clinicopathological characteristics

	CXCR4			CXCR5			CCR7		
Variables	Positive (*n* = 195)	Negative (*n* = 49)	*P*‐value	Positive (*n* = 155)	Negative (*n* = 89)	*P*‐value	Positive (*n* = 117)	Negative (*n* = 127)	*P*‐value
Sex, n (%)			0.581			0.129			0.018
Male	112 (57.4%)	26 (53.1%)		82 (52.9%)	56 (62.9%)		57 (48.7%)	81 (63.8%)	
Female	83 (42.6%)	23 (46.9%)		73 (47.1%)	33(37.1%)		60 (51.3%)	46 (36.2%)	
Median age (range), year	59 (37–74)	60 (39–74)	0.933	59 (37–74)	59 (39–74)	0.921	59 (39–74)	59 (37–74)	0.787
Median tumor size (range), cm	2.0 (1.1–3.0)	2.0 (1.2–3.0)	0.553	2.0 (1.1–3.0)	2.1 (1.2–3.0)	0.311	2.0 (1.2–3.0)	2.2 (1.1–3.0)	0.390
Tumor location, n (%)			0.711			0.287			0.310
RUL	63 (32.3%)	18 (36.7%)		48 (31.2%)	33 (37.5%)		37 (31.6%)	44 (34.6%)	
RML	10 (5.1%)	1 (2.0%)		6 (3.9%)	5 (5.7%)		3 (2.6%)	8 (6.3%)	
RLL	35 (17.9%)	7 (14.3%)		29 (18.8%)	13 (14.8%)		19 (16.2%)	23 (18.1%)	
LUL	57 (29.2%)	16 (32.7%)		47 (30.5%)	26 (29.5%)		37 (31.6%)	36 (28.3%)	
LLL	29 (14.9%)	6 (12.2%)		24 (15.6%)	11 (12.5%)		21 (17.9%)	14 (11.0%)	
Main bronchus	1 (0.5%)	1 (2.0%)		1 (0.6%)	1 (1.1%)		0 (0.0%)	2 (1.6%)	
N stage, n (%)			0.102			0.277			0.304
N_0_	139 (71.3%)	42 (85.7%)		110 (71.0%)	71 (79.8%)		83 (70.9%)	98 (77.2%)	
N_1_	24 (12.3%)	4 (8.2%)		19 (12.2%)	9 (10.1%)		13 (11.1%)	15 (11.8%)	
N_2_	32 (16.4%)	3 (6.1%)		26 (16.8%)	9 (10.1%)		21 (17.9%)	14 (11.0%)	
Histological type, n (%)			0.032			<0.001			<0.001
Adenocarcinoma	145 (74.4%)	44 (89.8%)		132 (85.2%)	57 (64.1%)		13 (11.1%)	37 (29.1%)	
Squamous cell carcinoma	46 (23.6%)	4 (8.2%)		20 (12.9%)	30 (33.7%)		104 (88.9%)	85 (66.9%)	
Others	4 (2.1%)	1 (2.0%)		3 (1.9%)	2 (2.2%)		0 (0.0%)	5 (3.9%)	
Tumor differentiation, n (%)			<0.001			<0.001			0.102
Well	20 (10.3%)	17 (34.7%)		12 (7.7%)	25 (28.1%)		16 (13.7%)	21 (16.5%)	
Moderate	110 (56.4%)	21 (42.9%)		93 (60.0%)	38 (42.7%)		71 (60.7%)	60 (47.2%)	
Poor	65 (33.3%)	11 (22.4%)		50 (32.3%)	26 (29.2%)		30 (25.6%)	46 (36.3%)	
Lymphovascular invasion, n (%)	84 (43.1%)	12 (24.5%)	0.017	69 (44.5%)	27 (30.3%)	0.030	32 (27.4%)	64 (50.2%)	<0.001
Visceral pleural invasion, n (%)	78 (40.0%)	13 (26.5%)	0.081	67 (43.2%)	24 (27.0%)	0.011	44 (37.6%)	47 (37.0%)	0.923
Recurrence, n (%)	67 (34.4%)	7 (14.3%)	0.006	54 (34.8%)	20 (22.5%)	0.043	94 (80.3%)	76 (59.8%)	0.001
Survival, n (%)	150 (76.9%)	46 (93.9%)	0.008	121 (78.1%)	75 (84.3%)	0.241	106 (90.6%)	90 (70.9%)	<0.001

CXCR4, CXCR5 and CCR7 expression levels were remarkably different in histological type (CXCR4, *P* = 0.032; CXCR5, *P* < 0.001; CCR7, *P* < 0.001) and LVI status (CXCR4, *P* = 0.017; CXCR5, *P* = 0.030; CCR7, *P* < 0.001). In addition, CXCR4 and CXCR5 expression levels were remarkably different in tumor differentiation (CXCR4, *P* < 0.001; CXCR5, *P* < 0.001). CXCR5 expression was significantly higher in tumors with visceral pleural invasion (*P* = 0.011), and CCR7 expression was significantly higher in females (*P* = 0.018). Further analyses revealed that LUAD had higher expression levels of CXCR4 and CXCR5, while LUSC had higher expression of CCR7. Positive CXCR4 and CXCR5 expression were more likely to have LVI, while positive CCR7 expression was correlated to a lower incidence of LVI. In addition, CXCR4 and CXCR5 expression levels were notably higher in moderately and poorly differentiated tumors compared with well differentiated tumors.

The expression levels of all the three chemokines were significantly different in recurrence (CXCR4, *P* = 0.006; CXCR5, *P* = 0.043; CCR7, *P* = 0.001), meanwhile CXCR4 and CCR7 expression were also significantly different in survival (CXCR4, *P* = 0.008; CCR7, *P* < 0.001). The data showed that CXCR4 and CXCR5 expression were higher in patients with recurrence, while CXCR4 expression was higher in patients who were deceased. However, CCR7 was found to have higher expression in patients without recurrence and patients who survived.

Apart from the results mentioned above, no significant differences were found in the expression of the chemokines in other clinicopathological characteristics of the patients.

### Survival analyses based on CXCR4, CXCR5 and CCR7 expression

**Figure 2:**
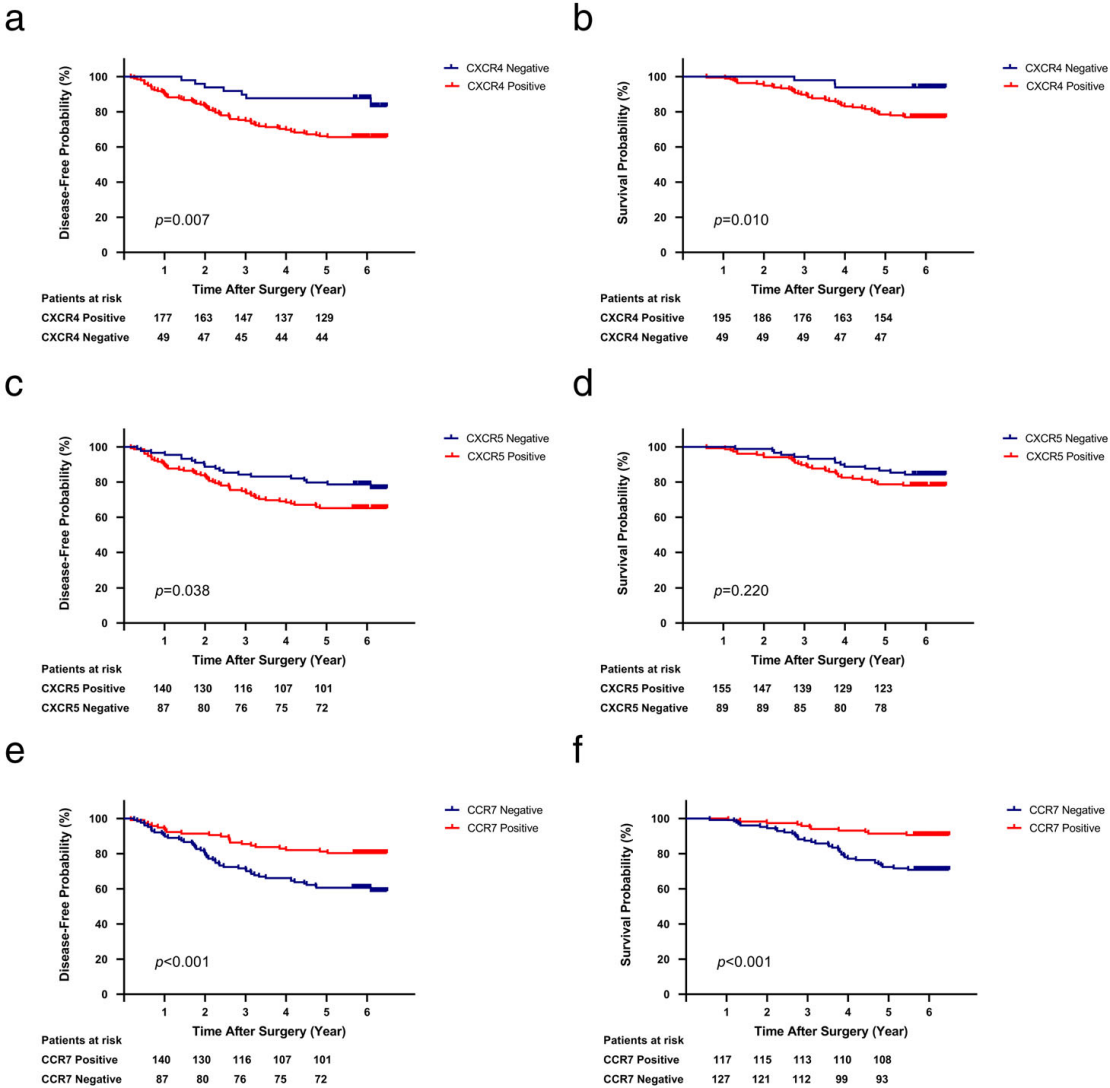
Survival outcomes based on CXCR4, CXCR5 and CCR7 expression. (**a**–**b**) DFS (**a**) and OS (**b**) of patients with positive/negative expression of CXCR4 (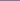) CXCR4 Negative; (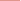) CXCR4 Positive, (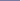) CXCR4 Negative; (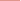) CXCR4 Positive. (**c**–**d**) DFS (**c**) and OS (**d**) of patients with positive/negative expression of CXCR5 (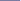) CXCR5 Negative; (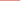) CXCR5 Positive, (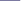) CXCR5 Negative; (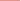) CXCR5 Positive. (**e**–**f**): DFS (**e**) and OS (**f**) of patients with positive/negative expression of CCR7 (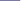) CCR7 Negative, (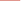) CCR7 Positive, (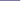) CCR7 Negative, (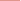) CCR7 Positive.

Survival outcomes based on CXCR4, CXCR5 and CCR7 expression were compared using the Kaplan‐Meier method (Fig. 2). Patients with positive CXCR4 expression had a significantly lower five‐year DFS (CXCR4 positive vs. CXCR4 negative, 66.2% vs. 87.8%, *P* = 0.007) and five‐year OS (CXCR4 positive vs. CXCR4 negative, 78.5% vs. 93.9%, *P* = 0.010) than those with negative CXCR4 expression. Subgroup analyses showed no significant difference in survival outcomes between patients with expression only on the membrane and patients with both membrane and nuclear expression (DFS: *P* = 0.731; OS: *P* = 0.847). Patients in the CXCR5 positive group had a significantly lower five‐year DFS than those in the CXCR5 negative group (CXCR5 positive vs. CXCR5 negative, 65.2% vs. 79.8%, *P* = 0.038), as well as a lower five‐year OS, but the difference was not significant (CXCR5 positive vs. CXCR5 negative, 78.7% vs. 86.5%, *P* = 0.220). Unlike the former two, patients with positive CCR7 expression had a significantly higher five‐year DFS (CCR7 positive vs. CCR7 negative, 81.2% vs. 60.6%, *P* < 0.001) and five‐year OS (CCR7 positive vs. CCR7 negative, 91.5% vs. 72.4%, *P* < 0.001). Subgroup analysis stratified by N stage demonstrated similar results in survival (CXCR4: *P* = 0.039, CXCR5: *P* = 0.522, CCR7: *P* < 0.001), and further analysis showed survival differentiated notably in N_0_ patients in the CXCR4 group (*P* = 0.030), and in N_0_ and N_2_ patients in the CCR7 group (*P* < 0.001; *P* = 0.030), indicating that patients of certain N stages who had positive CXCR4 or CCR7 expression had a significantly worse or better survival, which is consistent with the results in this study.

### Prognostic factors for survival of 244 patients with NSCLC

Univariate and multivariate analyses were conducted using the Cox proportional hazards regression model (Tables 4 and 5). The results showed that CXCR5 expression (HR: 2.698, 95% CI: 1.104–6.594, *P* = 0.030), CCR7 expression (HR: 0.454, 95% CI: 0.225–0.914, *P* = 0.027), N stage (HR: 2.939, 95% CI: 1.959–4.409, *P* < 0.001), tumor differentiation (HR: 3.561, 95% CI: 1.794–7.069, *P* < 0.001), and LVI (HR: 7.468, 95% CI: 3.083–18.089, *P* < 0.001) were the independent prognostic factors for DFS, while CCR7 expression (HR: 0.187, 95% CI: 0.069–0.508, *P* = 0.001), N stage (HR: 2.797, 95% CI: 1.609–4.862, *P* < 0.001), tumor differentiation (HR: 2.600, 95% CI: 1.090–6.198, *P* = 0.031), and LVI (HR: 4.640, 95% CI: 1.554–13.854, *P* = 0.006) were the independent prognostic factors for OS.

**Table 4:** Univariate analyses of prognostic factors for survival of 244 NSCLC patients

	Univariate analyses			
	DFS		OS	
Variable	HR (95% CI)	*P‐*value	HR (95% CI)	*P‐*value
Sex	0.852 (0.536–1.355)	0.499	0.669 (0.370–1.209)	0.184
Age	1.001 (0.975–1.028)	0.922	1.010 (0.977–1.044)	0.549
Tumor size	1.409 (0.916–2.167)	0.118	1.150 (0.675–1.957)	0.608
N stage	2.638 (2.042–3.407)	<0.001	2.496 (1.826–3.411)	<0.001
CXCR5 expression	1.709 (1.023–2.856)	0.041	1.473 (0.791–2.745)	0.223
CCR7 expression	0.428 (0.262–0.701)	0.001	0.290 (0.148–0.568)	<0.001
CXCR4 expression	2.770 (1.271–6.034)	0.010	4.142 (1.287–13.330)	0.017
Histological type	0.687 (0.419–1.127)	0.137	0.613 (0.336–1.118)	0.110
Tumor differentiation	4.269 (2.794–6.522)	<0.001	4.155 (2.435–7.088)	<0.001
Lymphovascular invasion	10.547 (5.781–19.241)	<0.001	7.089 (3.529–14.241)	<0.001
Visceral pleural invasion	1.176 (0.739–1.871)	0.494	0.827 (0.454–1.507)	0.535
Tumor location	0.879 (0.755–1.024)	0.099	0.913 (0.756–1.102)	0.342
Pathological subtype	2.069 (1.652–2.593)	<0.001	2.117 (1.583–2.830)	<0.001

**Table 5:** Multivariate analyses of prognostic factors for survival of 244 NSCLC patients

	Multivariate analyses			
	DFS		OS	
Variable	HR (95% CI)	*P‐*value	HR (95% CI)	*P*‐value
N stage	2.939 (1.959–4.409)	<0.001	2.797 (1.609–4.862)	<0.001
CXCR5 expression	2.698 (1.104–6.594)	0.030	1.510 (0.538–4.241)	0.434
CCR7 expression	0.454 (0.225–0.914)	0.027	0.187 (0.069–0.508)	0.001
CXCR4 expression	1.450 (0.531–3.962)	0.469	4.932 (0.968–25.133)	0.055
Tumor differentiation	3.561 (1.794–7.069)	<0.001	2.600 (1.090–6.198)	0.031
Lymphovascular invasion	7.468 (3.083–18.089)	<0.001	4.640 (1.554–13.854)	0.006
Pathological subtype	0.960 (0.702–1.313)	0.799	0.982 (0.657–1.467)	0.928

### Expression of CXCR4, CXCR5 and CCR7 in LUAD and LUSC in TCGA database

**Figure 3:**
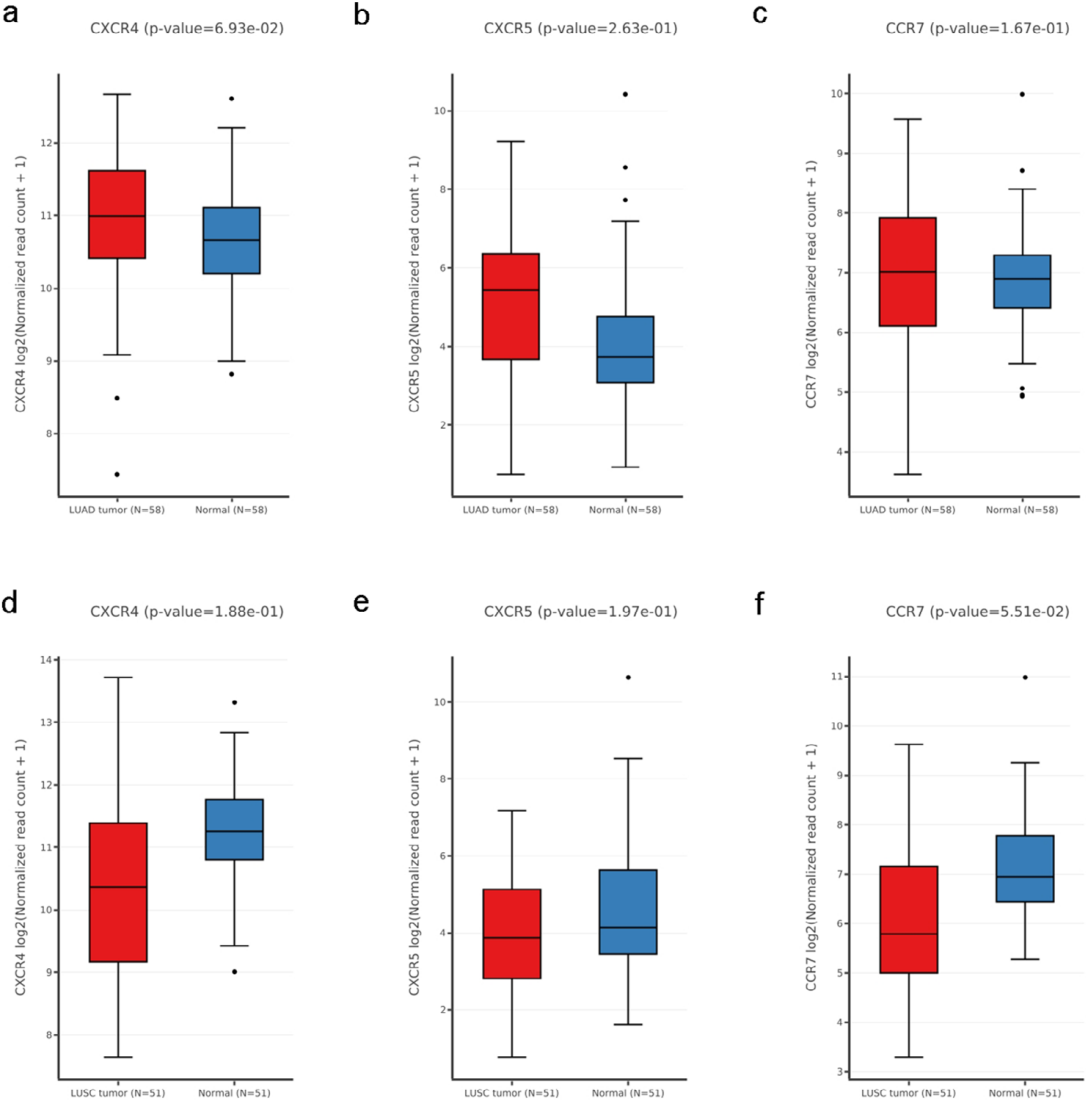
Expression of CXCR4, CXCR5 and CCR7 in LUAD and LUSC in TCGA database. (**a**–**c**) Expression of CXCR4 (**a**); CXCR5 (**b**); and CCR7 (c) in 58 LUAD cases (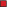) Tumor, (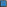) Normal, (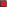) Tumor, (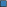) Normal, (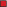) Tumor, (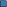) Normal. (**d**–**f**): Expression of CXCR4 (**d**), CXCR5 (**e**) and CCR7 (**f**) in 51 LUSC cases. LUAD, lung adenocarcinoma; LUSC, lung squamous cell carcinoma (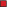) Tumor, (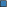) Normal, (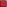) Tumor, (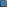) Normal, (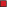) Tumor, (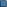) Normal.

The expression of CXCR4, CXCR5 and CCR7 in LUAD and LUSC in TCGA database are demonstrated in Fig 3. A total of 58 LUAD tumor tissues and their corresponding normal tissues were included. All three chemokines were overexpressed in tumor (Fig 3a–c), while no significant difference was found between tumor and normal tissues (CXCR4: *P* = 0.069, CXCR5: *P* = 0.263, CCR7: *P* = 0.167). A total of 51 LUSC tumor tissues and corresponding normal tissues were also analyzed (Fig 3d–f), and no significant difference was found (CXCR4: *P* = 0.188, CXCR5: *P* = 0.197, CCR7: *P* = 0.055).

## Discussion

Lung cancer is now the leading cause of cancer‐related death.^1^ Even for early stage patients, recurrence occurs in approximately 20% of individuals after surgery. Therefore, it is important to determine the prognostic factors to guide individualized precise treatments to improve survival outcomes.

Interactions between chemokines and their receptors have been reported to correlate to tumor dissemination, metastasis, tumor growth and cell survival.^43^ Several studies have shown that the overexpression of CXCR4 has been found in NSCLC and may be related to tumor progression and prognosis.^41^, ^44^ CXCR5 has been recently reported to play a role in the migration and invasion of NSCLC.^45^ Meanwhile, CCR7 has also been found to be involved in apoptosis, EMT, lymphangiogenesis, migration and invasion in NSCLC.^46^, ^47^, ^48^ However, the relationship between expression of CXCR4, CXCR5 and CCR7 and the survival outcomes in NSCLC remains to be clarified. In the current study, we demonstrated that expression of all three chemokines were significantly higher in tumor tissues using IHC. Meanwhile, expression of the three chemokines were correlated to histological type, tumor differentiation, LVI and survival; expression of CXCR4 and CCR7 were also correlated to recurrence; and CCR7 expression differed notably between sexes. Unlike the other two, CCR7 was highly expressed in LUSC and negatively correlated to LVI; in addition, patients with positive CCR7 expression had remarkably lower recurrence and higher survival rates. Survival analysis showed that CXCR4 and CXCR5 expression were correlated with worse DFS, and CXCR4 expression was also correlated with worse OS. On the contrary, CCR7 was an indicator of a better DFS and OS. Univariate and multivariate analyses were conducted and results demonstrated that CXCR5 expression, CCR7 expression, N stage, tumor differentiation and LVI were independent prognostic factors for DFS, while CCR7 expression, N stage, tumor differentiation and LVI were independent prognostic factors for OS. To ensure the accuracy of the results, we also compared the expression of all three chemokines in LUSC and LUAD cohorts from TCGA database, and found that the expressions of CXCR4, CXCR5 and CCR7 were higher in LUAD tumor tissues, which was consistent with our database, but the differences were not significant. In addition, the expression levels in LUSC were not significantly different, which might be due to insufficient samples in TCGA database.

CCR7 has a specific functional ligand, CCL19, which has been reported to have antitumor efficacy,^49^ and CCR7 itself has also been shown to be related to better prognoses.^50^ This relationship has also been validated in other types of tumors.^51^ CCR7 overexpression in NSCLC was found in both our study and TCGA database, and we also found that patients with CCR7 overexpression had significantly better survival outcomes. Our results are consistent with those in previous studies. However, controversies do exist. Some studies revealed that CCR7 promoted migration and invasion, inhibited apoptosis and interfered with the cell cycle, thus making it a biomarker for poor prognosis.^39^, ^42^ In addition, Gracio *et al*. demonstrated that a splicing imbalance of CCR7 was associated with the clinical outcomes of patients.^52^ All these studies indicate that CCR7 plays a complicated role in tumor progression and further studies are warranted to identify its actual function in NSCLC.

In conclusion, the present study demonstrated that the expression levels of CXCR4, CXCR5 and CCR7 were significantly higher in tumor tissues. Additionally, the expression of CXCR5 and CCR7 were independent prognostic factors for DFS, while CCR7 expression was an independent prognostic factor for OS. Moreover, all three chemokines were correlated to the survival outcomes of patients with clinical T1N0M0 NSCLC, providing potential prognosticators and therapy targets for lung cancer treatment.

## Disclosure

The authors declare no conflicts of interest.
